# (*E*)-3-[4-(Di­fluoro­meth­oxy)-3-hy­droxy­phen­yl]-1-phenyl­prop-2-en-1-one

**DOI:** 10.1107/S1600536813011288

**Published:** 2013-04-30

**Authors:** Thothadri Srinivasan, Govindaraj Senthilkumar, Kaliaperumal Neelakandan, Haridoss Manikandan, Devadasan Velmurugan

**Affiliations:** a Centre of Advanced Study in Crystallography and Biophysics, University of Madras, Guindy Campus, Chennai 600 025, India; b Department of Chemistry, Annamalai University, Annamalainagar 608 002, Tamilnadu, India

## Abstract

In the title compound, C_16_H_12_F_2_O_3_, the plane of the phenyl ring makes a dihedral angle of 3.22 (8)° with that of the benzene ring. The mol­ecule has an *E* conformation about the C=C bond. In the crystal, mol­ecules are linked *via* pairs of O—H⋯O hydrogen bonds, forming inversion dimers which are further consolidated by a pair of C—H⋯O hydrogen bonds. The dimers are linked *via* C—H⋯O hydrogen bonds, forming columns along the *b*-axis direction.

## Related literature

For the biological activity of chalcones, see: Di Carlo *et al.* (1999 ▶); Lin *et al.* (2002 ▶). For a related structure, see: Ranjith *et al.* (2010 ▶).
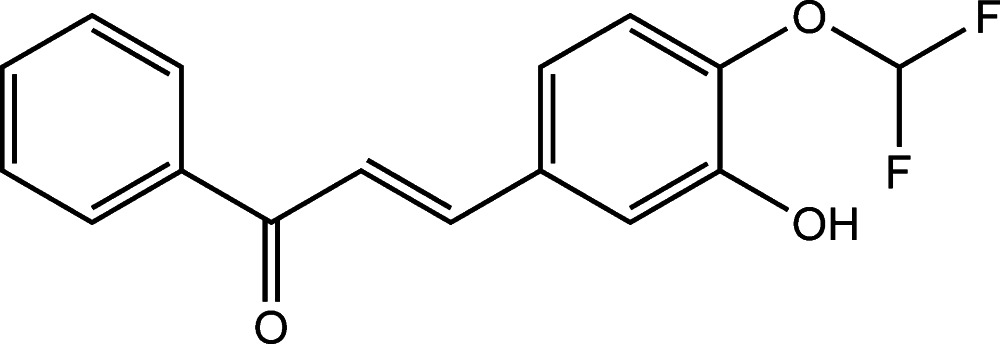


## Experimental

### 

#### Crystal data

C_16_H_12_F_2_O_3_
*M* *_r_* = 290.26
Monoclinic, 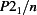

*a* = 17.1880 (11) Å
*b* = 4.1124 (3) Å
*c* = 19.6699 (13) Å
β = 106.289 (4)°
*V* = 1334.54 (16) Å^3^
*Z* = 4
Mo *K*α radiation
μ = 0.12 mm^−1^
*T* = 293 K
0.30 × 0.25 × 0.20 mm

#### Data collection

Bruker SMART APEXII area-detector diffractometer
Absorption correction: multi-scan (*SADABS*; Bruker, 2008 ▶) *T*_min_ = 0.966, *T*_max_ = 0.977
12412 measured reflections
3328 independent reflections
2456 reflections with *I* > 2σ(*I*)
*R*_int_ = 0.028

#### Refinement

*R*[*F* ^2^ > 2σ(*F* ^2^)] = 0.041
*wR*(*F* ^2^) = 0.120
*S* = 1.03
3328 reflections
191 parameters
H-atom parameters constrained
Δρ_max_ = 0.21 e Å^−3^
Δρ_min_ = −0.18 e Å^−3^

### 

Data collection: *APEX2* (Bruker, 2008 ▶); cell refinement: *SAINT* (Bruker, 2008 ▶); data reduction: *SAINT*; program(s) used to solve structure: *SHELXS97* (Sheldrick, 2008 ▶); program(s) used to refine structure: *SHELXL97* (Sheldrick, 2008 ▶); molecular graphics: *ORTEP-3 for Windows* (Farrugia, 2012 ▶); software used to prepare material for publication: *SHELXL97* and *PLATON* (Spek, 2009 ▶).

## Supplementary Material

Crystal structure: contains datablock(s) global, I. DOI: 10.1107/S1600536813011288/su2584sup1.cif

Structure factors: contains datablock(s) I. DOI: 10.1107/S1600536813011288/su2584Isup2.hkl

Supplementary material file. DOI: 10.1107/S1600536813011288/su2584Isup3.cml

Additional supplementary materials:  crystallographic information; 3D view; checkCIF report

## Figures and Tables

**Table 1:** Hydrogen-bond geometry (Å, °)

*D*—H⋯*A*	*D*—H	H⋯*A*	*D*⋯*A*	*D*—H⋯*A*
O2—H2⋯O3^i^	0.82	1.96	2.7722 (16)	172
C4—H4⋯O3^i^	0.93	2.48	3.1940 (19)	134
C1—H1⋯O1^ii^	0.98	2.40	3.3022 (19)	152

Symmetry codes: (i) 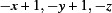; (ii) 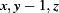.

**Fig. 1.**
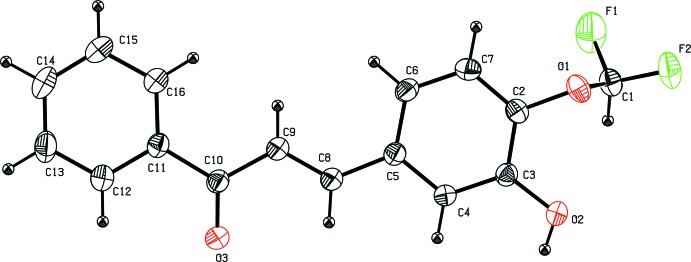
The molecular structure of the title molecule, with atom labelling. Displacement ellipsoids are drawn at the 30% probability level.

**Fig. 2.**
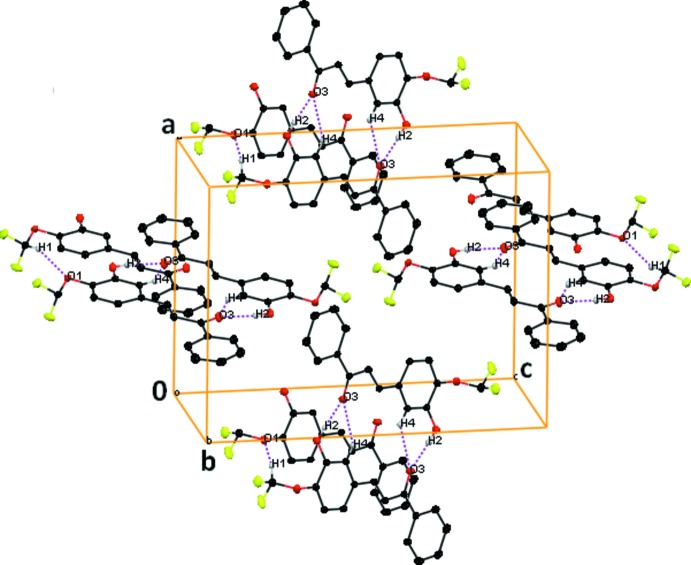
The crystal packing of the title compound viewed along the b axis. Hydrogen bonds are shown as dashed lines (see Table 1 for details; H-atoms not involved in hydrogen bonding have been omitted for clarity).

## supplementary crystallographic information

### Comment

Chalcones are a major classes of natural products with widespread distribution in fruits, vegetables, spices, tea and soy based foodstuff and have recently been the subject of great interest for their interesting pharmacological activities (Di Carlo *et al.*, 1999). Chalcones and flavonoids have been reported to be anti-tuberculosis agents (Lin *et al.*, 2002). Against this background and in order to obtain detailed information on molecular conformations in the solid state of similar compounds, we report herein on the synthesis and crystal structure of the title compound.

In the title compound, Fig. 1, the phenyl ring (C2-C7) makes a dihedral angle of 3.22 (8)° with the benzene ring (C11-C16). The hydroxyl oxygen atom, O2, deviates by -0.0243 (12) Å from the benzene ring mean plane.

In the crystal, molecules are linked via a pair of O-H···O hydrogen bonds forming inversion dimers which are further consolidated by a pair of C-H···O hydrogen bonds (Fig. 2 and Table 1). The dimers are linked via C-H···O hydrogen bonds forming columns along the b axis direction (Table 1).

### Experimental

A mixture of 3-hydroxy-4-difluoromethoxybezaldehyde (2 mmol), acetophenone (2 mmol) and sodiumhydroxide (2 mmol) in ethanol were placed in a conical flask and exposed to ultrasound irradiation. The reaction mixture was monitored by TLC. After completion of the reaction, the mixture was acidified with dilute HCl and kept in the fridge overnight. The product that separated out was washed with distilled water and recrystallized from ethanol [Yield = 96%; M.p. = 409-411 K]. Colourless block-like crystals, suitable for X-ray diffraction analysis, were obtained by slow evaporation of a solution of the title compound in hexane at room temperature.

### Refinement

H atoms were placed in calculated positions, with O-H = 0.82 Å and C—H = 0.93-0.98 Å, and refined in a riding model with *U*_iso_(H) = 1.5*U*_eq_(O) and = 1.2*U*_eq_(C).

### Crystal data

**Table:**

C_16_H_12_F_2_O_3_	*F*(000) = 600
*M**_r_* = 290.26	*D*_x_ = 1.445 Mg m^−^^3^
Monoclinic, *P*2_1_/*n*	Mo *K*α radiation, λ = 0.71073 Å
Hall symbol: -P 2yn	Cell parameters from 3328 reflections
*a* = 17.1880 (11) Å	θ = 1.4–28.3°
*b* = 4.1124 (3) Å	µ = 0.12 mm^−^^1^
*c* = 19.6699 (13) Å	*T* = 293 K
β = 106.289 (4)°	Block, colourless
*V* = 1334.54 (16) Å^3^	0.30 × 0.25 × 0.20 mm
*Z* = 4	

### Data collection

**Table:**

Bruker SMART APEXII area-detector diffractometer	3328 independent reflections
Radiation source: fine-focus sealed tube	2456 reflections with *I* > 2σ(*I*)
Graphite monochromator	*R*_int_ = 0.028
ω and φ scans	θ_max_ = 28.3°, θ_min_ = 1.4°
Absorption correction: multi-scan (*SADABS*; Bruker, 2008)	*h* = −22→21
*T*_min_ = 0.966, *T*_max_ = 0.977	*k* = −5→5
12412 measured reflections	*l* = −26→23

### Refinement

**Table:**

Refinement on *F*^2^	Primary atom site location: structure-invariant direct methods
Least-squares matrix: full	Secondary atom site location: difference Fourier map
*R*[*F*^2^ > 2σ(*F*^2^)] = 0.041	Hydrogen site location: inferred from neighbouring sites
*wR*(*F*^2^) = 0.120	H-atom parameters constrained
*S* = 1.03	*w* = 1/[σ^2^(*F*_o_^2^) + (0.0528*P*)^2^ + 0.3218*P*] where *P* = (*F*_o_^2^ + 2*F*_c_^2^)/3
3328 reflections	(Δ/σ)_max_ < 0.001
191 parameters	Δρ_max_ = 0.21 e Å^−^^3^
0 restraints	Δρ_min_ = −0.18 e Å^−^^3^

### Special details

**Table:**

Geometry. All esds (except the esd in the dihedral angle between two l.s. planes) are estimated using the full covariance matrix. The cell esds are taken into account individually in the estimation of esds in distances, angles and torsion angles; correlations between esds in cell parameters are only used when they are defined by crystal symmetry. An approximate (isotropic) treatment of cell esds is used for estimating esds involving l.s. planes.
Refinement. #========================================================================= # 8. Refinement Data #========================================================================= Refinement of F^2^ against ALL reflections. The weighted R-factor wR and goodness of fit S are based on F^2^, conventional R-factors R are based on F, with F set to zero for negative F^2^. The threshold expression of F^2^ > 2sigma(F^2^) is used only for calculating R-factors(gt) etc. and is not relevant to the choice of reflections for refinement. R-factors based on F^2^ are statistically about twice as large as those based on F, and R- factors based on ALL data will be even larger.

### Fractional atomic coordinates and isotropic or equivalent isotropic displacement parameters (Å^2^)

**Table:**

	*x*	*y*	*z*	*U*_iso_*/*U*_eq_	
C1	0.58013 (9)	0.9403 (4)	0.34909 (8)	0.0501 (4)	
H1	0.5553	0.7323	0.3305	0.060*	
C2	0.57744 (8)	1.0801 (3)	0.23416 (7)	0.0408 (3)	
C3	0.52043 (8)	0.8962 (3)	0.18585 (7)	0.0406 (3)	
C4	0.53484 (8)	0.8222 (4)	0.12164 (7)	0.0421 (3)	
H4	0.4969	0.7001	0.0885	0.051*	
C5	0.60481 (7)	0.9264 (3)	0.10571 (7)	0.0399 (3)	
C6	0.66097 (8)	1.1133 (4)	0.15554 (7)	0.0459 (3)	
H6	0.7078	1.1872	0.1457	0.055*	
C7	0.64685 (9)	1.1882 (4)	0.21944 (8)	0.0478 (3)	
H7	0.6843	1.3120	0.2527	0.057*	
C8	0.61704 (8)	0.8271 (4)	0.03816 (7)	0.0443 (3)	
H8	0.5763	0.6990	0.0094	0.053*	
C9	0.67854 (8)	0.8960 (4)	0.01260 (7)	0.0471 (3)	
H9	0.7203	1.0275	0.0388	0.057*	
C10	0.68243 (8)	0.7696 (4)	−0.05624 (7)	0.0434 (3)	
C11	0.75269 (8)	0.8529 (3)	−0.08315 (7)	0.0429 (3)	
C12	0.75121 (10)	0.7547 (4)	−0.15079 (8)	0.0587 (4)	
H12	0.7062	0.6441	−0.1785	0.070*	
C13	0.81561 (11)	0.8191 (5)	−0.17761 (9)	0.0662 (5)	
H13	0.8143	0.7488	−0.2229	0.079*	
C14	0.88160 (10)	0.9868 (5)	−0.13757 (10)	0.0655 (5)	
H14	0.9247	1.0326	−0.1560	0.079*	
C15	0.88416 (10)	1.0869 (5)	−0.07064 (10)	0.0710 (5)	
H15	0.9291	1.1997	−0.0435	0.085*	
C16	0.81987 (9)	1.0202 (4)	−0.04329 (8)	0.0576 (4)	
H16	0.8219	1.0885	0.0022	0.069*	
O1	0.56344 (6)	1.1715 (2)	0.29861 (5)	0.0490 (3)	
O2	0.45380 (6)	0.7923 (3)	0.20384 (5)	0.0547 (3)	
H2	0.4253	0.6830	0.1715	0.082*	
O3	0.62832 (6)	0.5935 (3)	−0.09091 (6)	0.0615 (3)	
F1	0.65995 (7)	0.9098 (4)	0.37665 (6)	0.1057 (5)	
F2	0.55249 (7)	1.0449 (3)	0.40166 (5)	0.0779 (3)	

### Atomic displacement parameters (Å^2^)

**Table:**

	*U*^11^	*U*^22^	*U*^33^	*U*^12^	*U*^13^	*U*^23^
C1	0.0568 (8)	0.0515 (8)	0.0449 (7)	0.0069 (7)	0.0190 (6)	−0.0019 (6)
C2	0.0461 (7)	0.0362 (7)	0.0401 (6)	0.0061 (6)	0.0120 (5)	−0.0010 (5)
C3	0.0368 (6)	0.0450 (7)	0.0406 (6)	0.0024 (6)	0.0120 (5)	0.0022 (6)
C4	0.0366 (6)	0.0507 (8)	0.0384 (6)	−0.0055 (6)	0.0097 (5)	−0.0029 (6)
C5	0.0374 (6)	0.0424 (7)	0.0404 (6)	0.0006 (5)	0.0116 (5)	0.0038 (5)
C6	0.0398 (7)	0.0487 (8)	0.0508 (8)	−0.0063 (6)	0.0153 (6)	0.0009 (6)
C7	0.0480 (7)	0.0440 (8)	0.0486 (7)	−0.0067 (6)	0.0090 (6)	−0.0057 (6)
C8	0.0409 (7)	0.0521 (8)	0.0406 (7)	−0.0057 (6)	0.0124 (5)	0.0000 (6)
C9	0.0429 (7)	0.0560 (9)	0.0441 (7)	−0.0083 (6)	0.0150 (6)	−0.0016 (6)
C10	0.0395 (7)	0.0498 (8)	0.0419 (7)	−0.0032 (6)	0.0128 (5)	0.0042 (6)
C11	0.0420 (7)	0.0457 (8)	0.0436 (7)	0.0005 (6)	0.0165 (5)	0.0058 (6)
C12	0.0577 (9)	0.0730 (11)	0.0498 (8)	−0.0128 (8)	0.0224 (7)	−0.0035 (8)
C13	0.0730 (11)	0.0793 (12)	0.0581 (9)	−0.0036 (10)	0.0377 (8)	0.0019 (9)
C14	0.0554 (9)	0.0719 (11)	0.0814 (12)	0.0000 (8)	0.0394 (9)	0.0111 (10)
C15	0.0479 (9)	0.0908 (14)	0.0791 (12)	−0.0185 (9)	0.0258 (8)	−0.0072 (10)
C16	0.0483 (8)	0.0727 (11)	0.0551 (8)	−0.0111 (8)	0.0200 (7)	−0.0068 (8)
O1	0.0638 (6)	0.0415 (5)	0.0433 (5)	0.0093 (5)	0.0178 (4)	−0.0050 (4)
O2	0.0452 (5)	0.0768 (8)	0.0471 (5)	−0.0116 (5)	0.0214 (4)	−0.0088 (5)
O3	0.0530 (6)	0.0835 (8)	0.0511 (6)	−0.0251 (6)	0.0199 (5)	−0.0131 (6)
F1	0.0658 (7)	0.1779 (15)	0.0725 (7)	0.0482 (8)	0.0180 (5)	0.0333 (8)
F2	0.0913 (8)	0.0979 (8)	0.0562 (6)	0.0185 (6)	0.0402 (5)	−0.0023 (5)

### Geometric parameters (Å, º)

**Table:**

C1—F2	1.3250 (16)	C8—H8	0.9300
C1—F1	1.3322 (18)	C9—C10	1.4693 (19)
C1—O1	1.3461 (18)	C9—H9	0.9300
C1—H1	0.9800	C10—O3	1.2244 (16)
C2—C7	1.378 (2)	C10—C11	1.4880 (17)
C2—C3	1.3830 (19)	C11—C16	1.383 (2)
C2—O1	1.4063 (16)	C11—C12	1.384 (2)
C3—O2	1.3589 (15)	C12—C13	1.379 (2)
C3—C4	1.3873 (18)	C12—H12	0.9300
C4—C5	1.3925 (17)	C13—C14	1.372 (3)
C4—H4	0.9300	C13—H13	0.9300
C5—C6	1.3974 (19)	C14—C15	1.368 (3)
C5—C8	1.4605 (18)	C14—H14	0.9300
C6—C7	1.380 (2)	C15—C16	1.385 (2)
C6—H6	0.9300	C15—H15	0.9300
C7—H7	0.9300	C16—H16	0.9300
C8—C9	1.3223 (18)	O2—H2	0.8200
			
F2—C1—F1	105.47 (13)	C8—C9—C10	121.50 (13)
F2—C1—O1	107.31 (12)	C8—C9—H9	119.3
F1—C1—O1	110.42 (14)	C10—C9—H9	119.3
F2—C1—H1	111.1	O3—C10—C9	119.92 (12)
F1—C1—H1	111.1	O3—C10—C11	120.23 (12)
O1—C1—H1	111.1	C9—C10—C11	119.85 (12)
C7—C2—C3	121.35 (12)	C16—C11—C12	118.51 (13)
C7—C2—O1	118.71 (12)	C16—C11—C10	122.99 (13)
C3—C2—O1	119.88 (12)	C12—C11—C10	118.50 (13)
O2—C3—C2	118.64 (12)	C13—C12—C11	120.78 (15)
O2—C3—C4	123.15 (12)	C13—C12—H12	119.6
C2—C3—C4	118.20 (12)	C11—C12—H12	119.6
C3—C4—C5	121.55 (12)	C14—C13—C12	120.00 (16)
C3—C4—H4	119.2	C14—C13—H13	120.0
C5—C4—H4	119.2	C12—C13—H13	120.0
C4—C5—C6	118.80 (12)	C15—C14—C13	120.14 (15)
C4—C5—C8	118.20 (12)	C15—C14—H14	119.9
C6—C5—C8	122.99 (12)	C13—C14—H14	119.9
C7—C6—C5	119.88 (12)	C14—C15—C16	120.01 (16)
C7—C6—H6	120.1	C14—C15—H15	120.0
C5—C6—H6	120.1	C16—C15—H15	120.0
C2—C7—C6	120.21 (13)	C11—C16—C15	120.55 (15)
C2—C7—H7	119.9	C11—C16—H16	119.7
C6—C7—H7	119.9	C15—C16—H16	119.7
C9—C8—C5	128.35 (13)	C1—O1—C2	114.90 (10)
C9—C8—H8	115.8	C3—O2—H2	109.5
C5—C8—H8	115.8		
			
C7—C2—C3—O2	178.98 (13)	C8—C9—C10—C11	179.99 (14)
O1—C2—C3—O2	−3.68 (19)	O3—C10—C11—C16	−172.66 (15)
C7—C2—C3—C4	0.0 (2)	C9—C10—C11—C16	6.6 (2)
O1—C2—C3—C4	177.37 (12)	O3—C10—C11—C12	6.5 (2)
O2—C3—C4—C5	−178.42 (13)	C9—C10—C11—C12	−174.29 (14)
C2—C3—C4—C5	0.5 (2)	C16—C11—C12—C13	0.7 (3)
C3—C4—C5—C6	−0.8 (2)	C10—C11—C12—C13	−178.48 (15)
C3—C4—C5—C8	177.55 (13)	C11—C12—C13—C14	−1.1 (3)
C4—C5—C6—C7	0.7 (2)	C12—C13—C14—C15	0.9 (3)
C8—C5—C6—C7	−177.60 (14)	C13—C14—C15—C16	−0.3 (3)
C3—C2—C7—C6	−0.2 (2)	C12—C11—C16—C15	−0.1 (3)
O1—C2—C7—C6	−177.53 (12)	C10—C11—C16—C15	179.00 (16)
C5—C6—C7—C2	−0.2 (2)	C14—C15—C16—C11	−0.1 (3)
C4—C5—C8—C9	−179.68 (15)	F2—C1—O1—C2	−170.69 (12)
C6—C5—C8—C9	−1.4 (3)	F1—C1—O1—C2	74.84 (16)
C5—C8—C9—C10	178.56 (14)	C7—C2—O1—C1	−101.79 (16)
C8—C9—C10—O3	−0.8 (2)	C3—C2—O1—C1	80.80 (16)

### Hydrogen-bond geometry (Å, º)

**Table:**

*D*—H···*A*	*D*—H	H···*A*	*D*···*A*	*D*—H···*A*
O2—H2···O3^i^	0.82	1.96	2.7722 (16)	172
C4—H4···O3^i^	0.93	2.48	3.1940 (19)	134
C1—H1···O1^ii^	0.98	2.40	3.3022 (19)	152

Symmetry codes: (i) −*x*+1, −*y*+1, −*z*; (ii) *x*, *y*−1, *z*.
